# Distinguishing Six Edible Berries Based on Metabolic Pathway and Bioactivity Correlations by Non-targeted Metabolite Profiling

**DOI:** 10.3389/fpls.2018.01462

**Published:** 2018-10-02

**Authors:** Dong Ho Suh, Eun Sung Jung, Gyu Min Lee, Choong Hwan Lee

**Affiliations:** ^1^Department of Bioscience and Biotechnology, Konkuk University, Seoul, South Korea; ^2^Department of Systems Biotechnology, Konkuk University, Seoul, South Korea

**Keywords:** berry, metabolomics, biosynthetic pathway, polyphenol, anti-oxidant activity

## Abstract

Berries have been used as valuable sources of polyphenols for human health; however, injudicious uses of berries are widespread without regard to the specific metabolite constituent of each berry. We classified 6 different edible berries (honeyberry, blueberry, mandarin melonberry, mulberry, chokeberry, and Korean black raspberry) based on their metabolite distributions in biosynthetic pathways by non-targeted metabolite profiling and bioactive correlation analysis. Principal component analysis revealed a distinct clustering pattern of metabolites for each berry. Metabolic pathway analysis revealed different biosynthetic routes of secondary metabolites in each berry. Mandarin melonberry contains a relatively higher proportion of genistein, genistein glycoside, and genistein-derived isoflavonoids and prenylflavonoids than the other berries. Various anthocyanin glycosides, synthesized from dihydroquercetin and cyanidin, were more abundant in chokeberry and honeyberry, whereas high levels of flavonoid-and anthocyanins-rutinoside forms were observed in Korean black raspberry. The levels of anthocyanins derived from dihydromyricetin were high in blueberry. The highest anti-oxidant activity was observed in chokeberry and Korean black raspberry, which is positively related to the proportional concentration of flavonoids, phenolics, and anthocyanins. The lowest sugar contents were observed in Korean black raspberry, highest acidity in honeyberry, and lowest acidity in mandarin melonberry, which were specific characteristics among the berries. Taken together, biosynthetic pathway and physicochemical characteristics analyses revealed that the different synthesized routes of flavonoids and anthocyanins and associated bio-activities may be distinct features in each berry and explain their phenotypic diversity at the molecular level.

## Introduction

Edible berries are among the most important sources of polyphenols (e.g., phenolic acids, flavonoids, anthocyanins, and tannins), which are closely associated with human health. Several studies have shown that many bioactive components found in berries have potent anti-oxidant, antimicrobial, anticancer, anti-inflammatory, and anti-neurodegenerative effects (Devore et al., 2012; Nile and Park, 2014). There are various edible berries, including blueberry, cranberry, bilberry, lingonberry, blackberry, red and black raspberry, cloudberry, red and black currants, honeysuckle berry, and black mulberry (Häkkinen et al., 1999; Mikulic-Petkovsek et al., 2012b). Although berries belong to many different genera such as *Lonicera, Vaccinium, Cudrania, Morus, Aronia*, and *Rubus*, many people consider berries to be a single type. While the main bio-active compounds in major berries have been examined, their metabolite profiles remain unclear. Recently, the use of berries has increased substantially; they are not only consumed as fresh fruits, but also utilized as extracts in processed products, including yogurts, fruit and vegetable beverages, jams, and dietary supplements (Clegg et al., 2011; Gironés-Vilaplana et al., 2012; Roopchand et al., 2013; Sun-Waterhouse et al., 2013; Nile and Park, 2014). Considering the rapid expansion of applications using berries and importance of their efficient use, it is crucial to understand their nutritional profiles.

Metabolomics has significantly advanced our fundamental understanding of the natural variance in metabolite composition between plants (Schauer and Fernie, 2006) and enabled biosynthesis of natural products derived from plants with bioactivity, which have potential applications for human health (Sumner et al., 2015). Particularly, a non-targeted metabolomics approach can be used for characterization and classification between plants based on differential or characteristic metabolites in the species (Díaz et al., 2016). Among them, metabolic pathway based non-targeted metabolomics has been used to study a wide variety of plants such as rice (Jung et al., 2013), pitayas (Suh et al., 2014), hot peppers (Jang et al., 2015), sorghums (Turner et al., 2016), and grape berry (Wang et al., 2017). The general genes and metabolites of flavonoid and anthocyanin biosynthetic pathway are discovered in model plants (Lepiniec et al., 2006), and the differences of anthocyanin production and distribution are regulated by genetic and environmental conditions (Wang et al., 2017). There is a lack of research on comparative analysis to reveal berry specific expression of genes or metabolites in biosynthetic pathway, even though each berry has different metabolic compositions and unique properties. Thus, the major objective of this study was to examine six different edible berries by determining their nutritional profiles based on metabolic pathway analysis to identify specific metabolic pathway markers vital for determining berry quality. Additionally, physicochemical characteristics and bioactivities were determined for each berry.

## Materials and methods

### Chemicals

Methanol, acetonitrile, and water were purchased from Fisher Scientific (Waltham, MA, USA). Diethylene glycol, gallic acid, naringin, methoxyamine hydrochloride, pyridine, *N*-methyl-*N*-(trimethylsilyl) trifluoroacetamide (MSTFA), 6-hydroxy-2,5,7,8- tetramethylchroman-2-carboxylic acid, hydrochloric acid, potassium persulfate, 2,2′-azinobis (3-ethylbenzothiazoline-6-sulfonic acid) diammonium salt (ABTS), hydrochloride, 2,4,6- tris(2-pyridyl)-trizine (TPTZ), iron(III) chloride hexahydrate, sodium acetate, acetic acid, sodium carbonate, sodium hydroxide, 1,1-diphenyl-2-picrylhydrazyl (DPPH), formic acid, and standard compounds were obtained from Sigma Chemical Co. (St. Louis, MO, USA).

### Sample information and preparation

Various batches of six species from six different genera of edible berries (3 batches for *Lonicera caerulea*, honeyberry, 5 for *Vaccinium corymbosum*, blueberry, 6 for *Cudrania tricuspidata*, mandarin melon berry, 6 for *Morus alba*, mulberry, 7 for *Aronia melanocarpa*, chokeberry, and 7 for *Rubus coreanus*, korean black raspberry) were purchased from a local farm. Detailed information regarding the harvest year and location of the corresponding berries is listed in Table 1. Each fresh berry was freeze-dried for 7 days and then ground into a fine powder with a mortar and pestle. Each powdered sample was stored at−20°C until metabolite extraction.

**Table 1:** Information of 6 kinds of edible berry samples.

**No**.	**Common name**	**Scientific name**		**Harvest year**	**Harvest location^a^ (Province)**	**Abbr^b^**
		**Family**	**Genus species**			
1	Honeyberry	Caprifoliaceae	*Lonicera caerulea*	2015	Chungcheongnam-do	L
2	Honeyberry	Caprifoliaceae	*Lonicera caerulea*	2015	Gangwon-do	
3	Honeyberry	Caprifoliaceae	*Lonicera caerulea*	2016	Chungcheongnam-do	
4	Blueberry	Ericaceae	*Vaccinium corymbosum*	2015	Gangwon-do	V
5	Blueberry	Ericaceae	*Vaccinium corymbosum*	2015	Gyeonggi-do	
6	Blueberry	Ericaceae	*Vaccinium corymbosum*	2015	Jeollabuk-do	
7	Blueberry	Ericaceae	*Vaccinium corymbosum*	2016	Jeollanam-do	
8	Blueberry	Ericaceae	*Vaccinium corymbosum*	2015	Gyeonggi-do	
9	Mandarin Melonberry	Moraceae	*Cudrania tricuspidata*	2015	Gyeongsangnam-do	C
10	Mandarin Melonberry	Moraceae	*Cudrania tricuspidata*	2015	Jeollabuk-do	
11	Mandarin Melonberry	Moraceae	*Cudrania tricuspidata*	2015	Jeollabuk-do	
12	Mandarin Melonberry	Moraceae	*Cudrania tricuspidata*	2015	Jeollanam-do	
13	Mandarin Melonberry	Moraceae	*Cudrania tricuspidata*	2015	Jeollabuk-do	
14	Mandarin Melonberry	Moraceae	*Cudrania tricuspidata*	2015	Gyeongsangnam-do	
15	Mulberry	Moraceae	*Morus alba*	2015	Gyeongsangbuk-do	M
16	Mulberry	Moraceae	*Morus alba*	2015	Jeollabuk-do	
17	Mulberry	Moraceae	*Morus alba*	2015	Gyeongsangbuk-do	
18	Mulberry	Moraceae	*Morus alba*	2015	Jeollabuk-do	
19	Mulberry	Moraceae	*Morus alba*	2015	Jeollanam-do	
20	Mulberry	Moraceae	*Morus alba*	2016	Jeollabuk-do	
21	Chokeberry	Rosaceae	*Aronia melanocarpa*	2015	Jeollabuk-do	A
22	Chokeberry	Rosaceae	*Aronia melanocarpa*	2015	Jeollabuk-do	
23	Chokeberry	Rosaceae	*Aronia melanocarpa*	2015	Jeollanam-do	
24	Chokeberry	Rosaceae	*Aronia melanocarpa*	2015	Chungcheongbuk-do	
25	Chokeberry	Rosaceae	*Aronia melanocarpa*	2015	Jeollabuk-do	
26	Chokeberry	Rosaceae	*Aronia melanocarpa*	2015	Gangwon-do	
27	Chokeberry	Rosaceae	*Aronia melanocarpa*	2015	Jeollabuk-do	
28	Korean Black Raspberry	Rosaceae	*Rubus coreanus*	2015	Jeollabuk-do	R
29	Korean Black Raspberry	Rosaceae	*Rubus coreanus*	2015	Jeollabuk-do	
30	Korean Black Raspberry	Rosaceae	*Rubus coreanus*	2015	Jeollabuk-do	
31	Korean Black Raspberry	Rosaceae	*Rubus coreanus*	2015	Jeollabuk-do	
32	Korean Black Raspberry	Rosaceae	*Rubus coreanus*	2015	Jeollabuk-do	
33	Korean Black Raspberry	Rosaceae	*Rubus coreanus*	2015	Jeollabuk-do	
34	Korean Black Raspberry	Rosaceae	*Rubus coreanus*	2015	Jeollabuk-do	

a *Harvest locations of 6 different kinds of berries were stated as province of Republic of Korea*.

b *Abbreviation*.

### Metabolite extraction

Each powdered sample (100 mg) was extracted twice with 1 mL of mixed solvent (methanol/water/HCL, 80/19.9/0.1, v/v) and 20 μL of internal standard (0.1 mg/mL, ampicillin) using a MM400 mixer mill (Retsch®; Haan, Germany) at a frequency of 30 s^−1^ for 10 min. After extraction, the extract was centrifuged at 12,578 *g* for 5 min at 4°C (Hettich Zentrifugen Universal 320, Tuttlingen, Germany). Supernatants were collected into a single e-tube and sieved through a 0.2-μm polytetrafluoroethylene filter, and then completely dried using a speed vacuum concentrator (Modulspin 31; BioTron, Inc., Bucheon-si, Korea). The dried samples were used for instrument analysis and bio-activity assays.

### UPLC-Q-TOF-MS and UHPLC-ESI-IT-MS/MS analysis

The dried extract samples were re-dissolved with mixed solvent for ultra-high-performance liquid chromatography linear trap quadrupole tandem mass spectrometry (UHPLC-LTQ-MS/MS) analyses and ultra-performance liquid chromatography-quadrupole-time of flight (UPLC-Q-TOF) MS. The final concentration of all analytes was 30 mg/mL. UHPLC-LTQ-MS/MS analysis was performed using a LTQ XLTM ion-trap mass spectrometer (Thermo Fisher Scientific) coupled with a DIONEX UHPLC system that included an UltiMate 3000 RS pump, UltiMate 3000 RS autosampler, UltiMate 3000 column compartment, and UltiMate 3000 variable wavelength detector (Dionex Corporation, Sunnyvale, CA, USA). Chromatographic separation was performed on a Syncronis C18 column (100 × 2.1 mm, 1.7 μm particle size; Thermo Fisher Scientific) and the injection volume was 10 μL. The flow rate was 0.3 mL/min and column temperature was 40°C. The mobile phase consisted of 0.1% formic acid in water (solvent A) and 0.1% formic acid in acetonitrile (solvent B). The initial condition was 5% of solvent B for 1 min, which was increased to 65% of solvent B over 14 min and increased to 100% of solvent B over 3 min, and then isothermally held for 3 min. The system was returned to the initial condition over a gradient and maintained for 3 min. The total run time was 25 min. Mass spectra were acquired over the range of *m/z* 100–1,000 in both negative and positive ion modes. The source voltage was ±5 kV, collision voltage was 39 V, and capillary temperature was 275°C. Tandem mass spectrometry analysis was conducted by turbo data-dependent scan-type scanning under conditions identical to those used for MS scanning. Quality control samples were analyzed after every 10 samples to obtain reliable MS data.

For UPLC-Q-TOF-MS analysis, a Waters ACQUITY UPLC system (Waters Corp., Milford, MA, USA) equipped with a binary solvent delivery system, auto-sampler, and UV detector was used. Chromatographic separation was performed on a Waters ACQUITY BEH C18 column (i.d., 100 × 2.1 mm, 1.7 μm particle size) and the injection volume was 5 μL. The column temperature was 37°C and flow rate was 0.3 mL/min. The mobile phase consisted of 0.1% v/v formic acid in water (A) and 0.1% v/v formic acid in acetonitrile (B). The initial condition was 5% B for 1 min, which was then linearly increased to 100% B over 9 min. The total run time was 14 min, including re-equilibration of the column to the initial condition. For MS experiments, Waters Q-TOF Premier MS (Micromass MS Technologies, Manchester, UK) was operated in both negative and positive ion modes with an *m/z* range of 100–1,000. The source temperature was 100°C, collision energy was 10 eV, and collision gas flow was 0.3 mL/min. The desolvation gas was 650 L/h at a temperature of 300°C. The capillary voltage and sample cone voltage were 2.5 kV and 50 V, respectively. The V mode was used for the mass spectrometer, and data were collected in centroid mode with a scan accumulation of 0.2 s. Leucine encephalin was used as the reference lock mass (*m/z* 554.2615) by independent LockSpray interference.

### Data processing and multivariate statistical analysis

UHPLC-LTQ-MS data were acquired with Xcalibur software (version 2.00, Thermo Fisher Scientific) and converted into netCDF format (^*^.cdf) using the same Xcalibur software, Peak detection, retention time correction, and alignment were conducted using the MetAlign software package (http://www.metalign.nl). The resulting data were exported to an Excel spreadsheet (Microsoft; Redmond, WA, USA). Multivariate statistical analysis was performed using SIMCA-P+ 12.0 software (Umetrics; Umeå, Sweden). The data sets were auto-scaled (unit variance scaling) and mean-centered in a column-wise fashion. The significantly different metabolites between berries were selected based on variable importance projection (VIP) values, and significance was tested by analysis of variance (ANOVA) and Duncan's multiple range tests using PASW Statistics 18 software (SPSS, Inc., Chicago, IL, USA). Selected metabolites were tentatively identified by comparing mass spectra, retention time, mass fragment patterns, UV absorbance, and elemental compositions derived from UHPLC-LTQ-IT-MS/MS and UPLC-Q-TOF-MS analyses considering standard compounds, in-house library, and published references. The heatmap and correlation map were constructed by MeV software (http://www.tm4.org/).

### Determination of antioxidant activity by ABTS, DPPH, and FRAP

Three antioxidant activity tests, including ABTS, DPPH, and ferric reduction ability of plasma (FRAP), were performed using slightly modified procedures described previously (Jung et al., 2013). For the ABTS assay, 7 mM ABTS was dissolved in 2.45 mM potassium persulfate solution and the sample was incubated in the dark for 1 day at room temperature to obtain a dark-blue colored solution. The solution was then diluted until the absorbance reached 0.7 ± 0.02 at 734 nm using a microplate reader (Spectronic Genesys 6, Thermo Electron, Waltham, mA, USA). Twenty microliters of sample were then mixed with 180 μL of diluted ABTS solution in 96-well plates and allowed to react for 6 min in the dark. Absorbance was measured at 734 nm using a microplate reader.

For the DPPH assay, 20 μL of sample extract was mixed with 180 μM of 0.2 mM DPPH ethanol solution in 96-well plates for 20 min at room temperature. Absorbance was measured at 515 nm using a microplate reader.

The FRAP assay was conducted with freshly prepared FRAP reagent, which was made by combining mixed acetate buffer (pH 3.6), 10 mM TPTZ (in 40 mM HCl solution), and 20 mM FeCl3•6H2O (in distilled water) in a ratio of 10:1:1, respectively. Ten microliters of each sample were then mixed with 300 μL of FRAP reagent in 96-well plates for 6 min at 37°C. The absorbance was measured at 570 nm using a microplate reader. All results were presented as the Trolox equivalent antioxidant capacity (mM), with the standard solution concentration curve ranging from 0.0078 to 1.000 mM, and all experiments were carried out in triplicate.

### Determination of total phenolic, flavonoid, and anthocyanin contents

Total phenolic content was measured using the Folin-Ciocalteu colorimetric method. Briefly, 100 μL of 0.2N Folin-Ciocalteu's phenol reagent was added to 20 μL of each sample in 96-well plates. After incubation in the dark for 5 min, 80 μL of 7.5% sodium carbonate solution was added to the mixture, which was then incubated in the dark for an additional 60 min at room temperature. The absorbance was measured at 750 nm using a microplate reader (Spectronic Genesys 6). The results were presented as gallic acid equivalent concentration (ppm), and the standard solution concentration curve ranged from 1.9531 to 500 ppm.

To analyze total flavonoid content, 180 μL of 90% diethylene glycol (in distilled water), 20 μL of 1N NaOH, and 20 μL of each sample were mixed in 96-well plates, and then incubated in the dark for 60 min at room temperature. The absorbance was measured at 405 nm using a microplate reader. The results were presented as the naringin equivalent concentration (ppm), and the standard solution concentration curve ranged from 0.7813 to 200 ppm.

To measure the total anthocyanin content, two buffer systems, potassium chloride (pH 1.0) and sodium acetate (pH 4.5), were analyzed by the pH differential method using a microplate reader. Twenty microliters of sample were mixed with 180 μL of corresponding buffer, with the resulting solution was read against a blank at 515 and 750 nm. Absorbance was calculated as: *A* = (*A*515–*A*750 nm) pH 1.0 – (*A*515–*A*750 nm) pH 4.5. The concentration of monomeric anthocyanin pigment was expressed as milligrams of cyanidin-3-glucoside and calculated as:

$$\begin{array}{l} \text{Monomeric anthocyanin pigment }\left(\text{mg}/\text{L}\right)\\ \;=\text{A }\times \text{ Mr }\times \text{ DF }\times \;1000\;÷\left(\text{ϵ }\times \;1\right) \end{array}$$

where *A* is the absorbance, *Mr* is the molecular weight (449.2), DF is the dilution factor, and ε is the molar absorptivity (269,000). All experiments were conducted in triplicate.

### Determination of physiochemical characters-sugar content, titratable acidity, and pH

Fresh berry fruits were squeezed using gauze to obtain the fresh juice extract. The sugar contents (100 μL of each fresh juice extract) were measured using a sugar meter (HANNA Instruments, Woonsocket, RI, USA). Each juice extract (3 mL) was diluted with distilled water (27 mL) and the pH was measured using a pH meter (Thermo Fisher). Titratable acidity was estimated by titrating the fresh juice extracts with 0.1N NaOH solution to a pH of 8.4. All experiments were carried out in triplicate.

## Results

### Non-targeted metabolite profiling of 6 different edible berries

**Figure 1:**
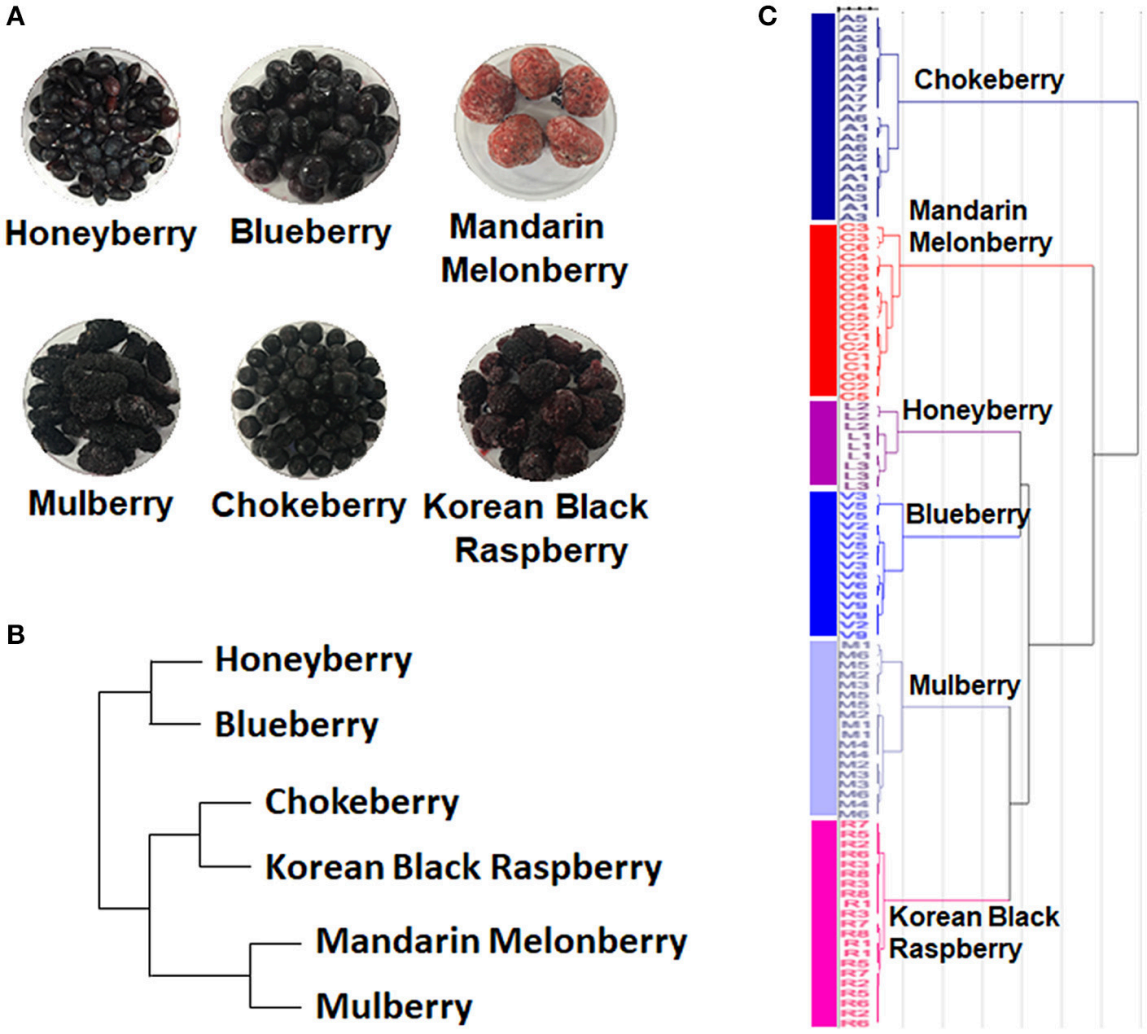
Photographs of the fruits **(A)** and results of taxonomic classification based on gene **(B)** and metabolites **(C)** in 6 different kinds of edible berries. Taxonomic classification based on gene results on a NCBI taxonomy database and metabolite results of metabolite profiling using UHPLC-LTQ-IT-MS/MS.

The 6 edible berries showed quite different morphological features, i.e., shape, color, and size (Figure 1A). To compare the taxonomic classification between gene- and metabolite-based analysis among 6 different edible berries, we constructed a phylogenetic tree based on the National Center for Biotechnology Information (NCBI) taxonomy database (Figure 1B) and chemotaxonomic dendrogram by hierarchical cluster analysis derived from LC-MS based metabolite profiling (Figure 1C). According to phylogenetic tree, there were 2 characteristic branches; honeyberry and blueberry, and other berry genera. Other berry genera were also divided depending on their family. Mandarin melonberry and mulberry, which belong to the family Moraceae, were clustered and distinguished from the Rosaceae family, which includes chokeberry and Korean black raspberry (Table 1). The results of chemotaxonomic classification derived from LC-MS metabolite profiling showed different patterns from the phylogenetic tree (Figure 1C). The first branch was divided into chokeberry and other berry genera, and these berry genera were divided into mandarin melonberry and the remaining 4 berry genera. Honeyberry and blueberry were clustered and distinguished from the sub-branch of mulberry and Korean black raspberry. Both taxonomic trees were well-separated by each genus but showed different patterns.

**Figure 2:**
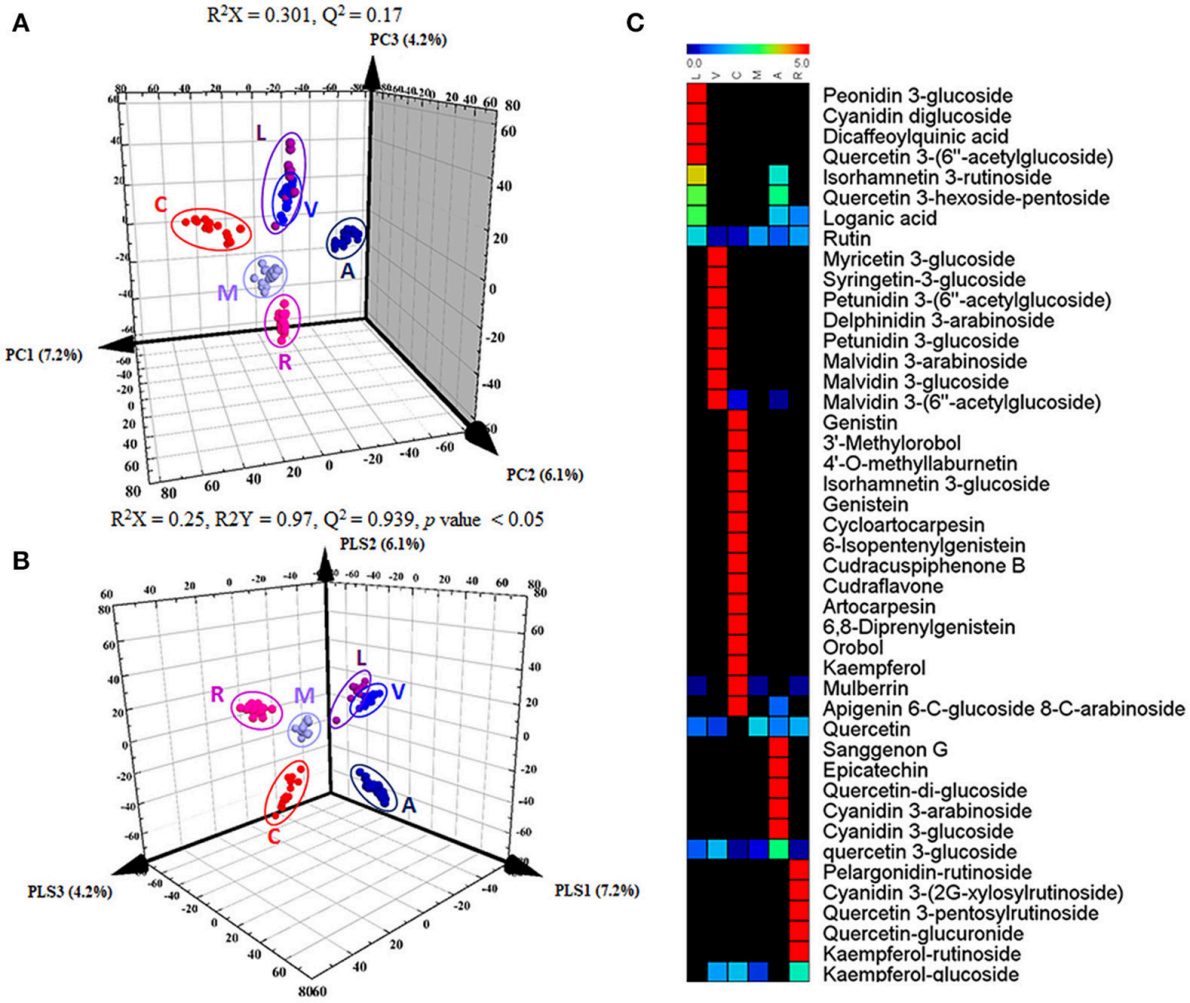
Results of 3D-PCA score plot **(A)**, 3D-PLSDA score plot **(B)**, and heat map analysis **(C)** in 6 different kinds of edible berries derived from UHPLC-LTQ-IT-MS/MS data. In heat map analysis, significantly differed metabolites were determined (VIP > 1.0 and I value < 0.05). L, honeyberry; V, blueberry; C, mandarin melonberry; M, mulberry; A, chokeberry; R, Korean black raspberry.

To distinguish differences in metabolites among the 6 different edible berries (total of 34 berries), non-targeted metabolite profiling was performed by UHPLC-ESI-IT-MS/MS combined with multivariate analysis. The principle component analysis (PCA) score plot (Figure 2A) revealed that all 6 edible berries differed from each other and clustered depending on their genus rather than various environment factors, and showed similar results in the partial least squares-discriminant analysis score plot (Figure 2B). The metabolites significantly contributing to discrimination among berries were selected based on the VIP value (>1.0) from partial least squares-discriminant analysis and *p* value (< 0.05) from one-way ANOVA. Although berry samples were collected from different locations at various time (Table 1), the PCA score plot showed that metabolic differences between berry samples mostly depended on their phylogeny rather than on other factors. A total of 51 metabolites were identified, of which only 44 significantly differed between the 6 different edible berries (Table 2). These metabolites were 12 anthocyanins (4 cyanidin derivatives, 2 petunidin derivatives, 3 malvidin derivatives, delphinidin 3-arabinoside, pelargonidin-rutinoside, and peonidin 3-glucoside), 29 flavonoids (3 kaempferol derivatives, 2 genistein derivatives, 10 quercetin derivatives, 2 myricetin derivatives, 5 isoflavonoids, 4 prenylated flavonoids, apigenin 6-C-glucoside 8-C-arabinoside, epicatechin, and sanggenon G), and 3 miscellaneous metabolites. Significantly different metabolites among the 6 different edible berries were visualized by heat map analysis (Figure 2C). In honeyberry, the levels of several anthocyanins (peonidin 3-glucoside and cyanidin diglucoside), 4 quercetin derivatives, dicaffeoylquinic acid, and loganic acid were higher than those in other berries, while many types of anthocyanins were high in blueberry. The flavonoid contents, particularly isoflavonoids and prenylated flavonoids, were significantly high in mandarin melonberry. Chokeberry and Korean black raspberry contained different flavonoids. The results of non-targeted metabolite profiling showed that berries had different quantities and varieties of metabolites, particularly flavonoids and anthocyanins, and further information is needed to evaluate the characteristic phenotypes of each berry genus.

**Table 2:** Differential metabolites in 6 kinds of edible berries derived from the UHPLC-LTQ-IT-MS/MS and UPLC-Q-TOF-MS analyses.

**No**.	**Tentative identifications**	**VIP^a^ 1**	**VIP 2**	**VIP 3**	**RT (min)^b^**	**MW^c^**	**Measured mass**		**MS^n^ fragments (m/z)**	**UV λmax (nm)**	**m/z**	**Molecular formula**	**mDa**	**PPM**	**DBE**	**i-Fit (norm)**
							**m/z**	**Adducts**								
***Anthocyanins***																
1	Cyanidin diglucoside^*^	0.17	0.78	1.07	1.26	611	611	M+	611, 576, 449, 287	280, 367, 516	611.1674	C27H31O16	5.2	8.5	12.5	3.3
2	Cyanidin 3-(2G-xylosylrutinoside)^*^	0.62	1.58	2.35	1.27	727	727	M+	727, 581, 433, 287	277, 523	727.2023	C32H39O19	-6.3	-8.7	13.5	1.6
3	Cyanidin 3-rutinoside	-	-	-	1.35	595	595	M+	595, 577, 449, 287	281, 520	595.1573	C27H31O15	-4.4	-7.4	12.5	0.5
4	Cyanidin 3-arabinoside^*^	1.92	1.46	1.21	1.87	419	419	M+	419, 401, 377, 329, 287	280, 516	419.0957	C20H19O10	-2.1	-5.0	11.5	0.2
5	Cyanidin 3-glucoside^*^	1.93	1.54	1.29	1.99	449	449	M+	449, 431, 287	280, 517	449.1097	C21H21O11	1.3	2.9	11.5	3.9
6	Delphinidin 3-glucoside	-	-	-	1.27	465	465	M+	465, 333, 303	277, 523	465.1078	C21H21O12	-4.0	-8.6	12.5	1.1
7	Delphinidin 3-arabinoside^*^	0.21	0.70	1.91	1.32	435	435	M+	435, 421, 392, 303, 273	277, 523	435.0963	C20H19O11	3.6	8.3	11.5	1.0
8	Petunidin 3-glucoside^*^	0.20	0.71	1.92	1.57	479	479	M+	479, 461, 334, 317	239, 279, 523	479.1231	C22H23O12	4.1	8.6	11.5	1.3
9	Petunidin 3-(6”-acetylglucoside)^*^	0.13	0.45	1.21	8.89	521	521	M+	521, 317, 302, 274	276, 526	521.1253	C24H25O13	-4.2	-8.1	12.5	4.0
10	Pelargonidin-rutinoside^*^	0.64	1.57	2.30	7.77	579	577	[M-H]^−^	579, 433, 271, 243, 225, 215	280, 516	577.1595	C27H29O14	3.8	6.6	13.5	3.6
11	Peonidin 3-glucoside^*^	0.24	0.82	1.50	7.97	463	463	M+	463, 301, 286	234, 516	463.1268	C22H23O11	2.8	6.0	11.5	2.6
12	Malvidin 3-glucoside^*^	0.20	0.70	1.90	7.94	493	493	M+	493, 475, 461, 451, 361, 331	279, 520	493.1306	C23H25O12	-4.0	-8.1	11.5	4.3
13	Malvidin 3-arabinoside^*^	0.21	0.75	2.03	8.25	463	463	M+	463, 331, 301	278, 527	463.1304	C22H23O11	4.3	9.3	11.5	0.2
14	Malvidin 3-(6”-acetylglucoside)^*^	0.21	0.40	1.25	9.40	535	535	M+	535, 517, 331	353, 528	535.1418	C25H27O13	-3.4	-6.4	12.5	2.5
***Flavonoids***																
15	Kaempferol-rutinoside^*^	0.65	0.87	1.14	7.42	594	593	[M-H]^−^	593, 575, 547, 467, 357, 339, 327, 309, 299, 285	280, 327	593.1448	C27H29O15	-5.8	-9.8	13.5	2.7
16	Kaempferol-glucoside^*^	0.64	0.48	1.19	10.44	448	447	[M-H]^−^	447, 357, 327,299, 285	265, 320	447.0956	C21H19O11	2.9	6.5	12.5	1.0
17	Genistein-diglucoside	0.59	0.58	0.67	9.99	594	593	[M-H]^−^	593, 575, 503, 485, 473, 383, 353	287,318, 366	593.1513	C27H29O15	0.7	1.2	13.5	0.6
18	Genistin^*^	0.42	1.57	1.29	10.50	432	431	[M-H]^−^	431, 413, 387, 311, 269	264, 316	431.0965	C21H19O10	-1.3	-3.0	12.5	0.7
19	Genistein^*^	1.54	2.15	1.76	13.59	270	269	[M-H]^−^	269, 241, 225, 201, 181	217	269.0442	C15H9O5	-0.8	-3.0	11.5	0.5
20	Isorhamnetin 3-glucoside^*^	0.53	1.94	1.60	8.28	478	477	[M-H]^−^	477, 445, 401, 385, 314, 299	290, 316, 366	477.103	C22H21O12	-0.1	-0.2	12.5	0.4
21	Isorhamnetin 3-rutinoside^*^	0.94	0.70	1.16	10.14	624	623	[M-H]^−^	623, 608, 431, 315, 300, 271	327	623.1619	C28H31O16	0.7	1.1	13.5	0.8
22	Quercetin-di-glucoside^*^	2.73	1.94	1.58	8.72	626	625	[M-H]^−^	625, 607, 579,5 05, 463, 445, 343, 301, 271, 255	280, 366	625.1343	C27H29O17	-6.2	-9.9	13.5	2.3
23	Quercetin 3-pentosylrutinoside^*^	0.72	0.97	1.27	8.79	742	741	[M-H]^−^	741, 723, 609, 591, 573, 475, 355, 301, 271	279, 366	741.1893	C32H37O20	1.5	2.0	14.5	1.6
24	Quercetin 3-hexoside-pentoside^*^	2.67	1.92	1.67	9.24	596	595	[M-H]^−^	595, 577, 343, 301, 271	273, 366	595.1187	C26H27O16	0.4	0.7	13.5	1.5
25	Rutin^*^	0.01	1.57	1.52	9.46	610	611	[M+H]^+^	611, 465, 449, 303	269, 329, 366	611.1609	C27H31O16	-0.3	-0.5	12.5	3.8
26	Quercetin-glucuronide^*^	0.65	1.66	2.45	9.82	478	477	[M-H]^−^	477, 315, 301, 179	366	477.0686	C21H17O13	1.7	3.6	13.5	0.9
27	quercetin 3-glucoside^*^	2.56	1.84	2.22	9.85	464	463	[M-H]^−^	463, 343, 301, 264	279, 320	463.0868	C21H19O12	-0.9	-1.9	12.5	0.3
28	Quercetin 3-(6”-acetylglucoside)^*^	0.52	0.51	1.41	10.55	506	505	[M-H]^−^	505, 463, 445, 343, 301	280	505.0965	C23H21O13	-0.5	-1.0	13.5	1.7
29	Quercetin^*^	0.34	1.06	1.33	12.53	302	301	[M-H]^−^	303, 285,257, 229, 165, 137, 121, 111	218, 279, 366	301.0341	C15H9O7	-0.7	-2.3	11.5	1.3
30	Apigenin 6-C-glucoside 8-C-arabinoside^*^	0.06	1.50	1.24	8.86	564	563	[M-H]^−^	563, 545, 503, 473, 443, 425, 383, 353	259,366	563.1396	C26H27O14	-0.5	-0.9	13.5	0.5
31	Myricetin 3-glucoside^*^	0.54	0.49	1.79	9.03	480	479	[M-H]^−^	461, 359, 317, 179	278,378	479.0814	C21H19O13	-1.2	-2.5	12.5	0.5
32	Epicatechin^*^	3.51	2.66	2.21	9.85	290	289	[M-H]^−^	291, 273, 245, 227, 201, 184, 159	269, 345	289.0706	C15H13O6	-0.6	-2.1	9.5	0.7
33	Syringetin 3-glucoside^*^	0.59	0.54	1.77	10.53	508	507	[M-H]^−^	507, 479, 464, 417, 387, 359, 345, 329, 302	264,326,366	507.1111	C23H23O13	-2.8	-5.5	12.5	0.2
34	Kaempferol^*^	1.76	2.44	2.01	12.10	286	287	[M+H]^+^	287, 269, 259, 241, 231, 153	279	287.0574	C15H11O6	1.8	6.3	10.5	0.7
35	Orobol^*^	0.53	1.97	1.62	12.24	286	285	[M-H]^−^	285, 257, 241, 229, 217	266	285.0399	C15H9O6	-0.1	-0.4	11.5	0.3
36	3'-Methylorobol^*^	0.51	1.90	1.57	13.72	300	299	[M-H]^−^	299, 284, 263, 253	258	299.0556	C16H11O6	0.0	0.0	11.5	0.4
37	Sanggenon G^*^	2.83	2.02	1.64	11.21	694	693	[M-H]^−^	693, 663, 644, 613, 563, 547, 501, 416,401,269	280	693.2404	C40H37O11	6.8	9.8	22.5	4.8
38	Artocarpesin^*^	0.51	1.90	1.56	16.84	354	353	[M-H]^−^	353, 325, 309, 298, 284, 219	269,345	353.1028	C20H17O6	0.3	0.8	12.5	0.4
39	Alpinumisoflavone	0.24	0.90	0.74	18.21	336	335	[M-H]^−^	335, 317, 289, 247, 221, 173	266	335.0916	C20H15O5	-0.3	-0.9	13.5	0.1
40	6-Isopentenylgenistein^*^	0.43	1.57	1.29	18.25	338	337	[M-H]^−^	337, 322, 293, 282, 269	266	337.1059	C20H17O5	-1.7	-5.0	12.5	0.6
41	4'-O-methyllaburnetin^*^	0.47	1.73	1.42	18.52	368	367	[M-H]^−^	367, 352, 309, 298, 219	269, 339	367.1178	C21H19O6	-0.4	-1.1	12.5	0.2
42	Cudraflavone^*^	0.50	1.95	1.60	20.03	422	421	[M-H]^−^	421, 393, 378, 366, 323, 311, 299, 269	223, 281	421.1639	C25H25O6	-1.2	-2.8	13.5	0.7
43	Cycloartocarpesin^*^	0.52	1.93	1.59	20.28	352	351	[M-H]^−^	351, 336, 296	223, 281	351.124	C21H19O5	0.8	2.3	12.5	0.2
44	6,8-Diprenylgenistein^*^	0.55	2.00	1.64	20.89	406	405	[M-H]^−^	405, 390, 377, 350, 321, 307, 295,2 43	221, 269, 334	405.1712	C25H25O5	1.0	2.5	13.5	0.2
***Etc***.																
45	Mulberrin^*^	1.49	1.97	1.62	20.02	422	423	[M+H]^+^	423, 405, 367, 311	222, 280	423.179	C25H27O6	-1.8	-4.3	12.5	3.1
46	Chlorogenic acid	0.25	0.82	0.75	6.17	354	353	[M-H]^−^	353, 191, 179, 173	294, 325	353.0872	C16H17O9	-0.1	-0.3	8.5	6.5
47	Alpigenoside	0.44	0.35	0.30	7.98	436	435	[M-H]^−^	435, 388, 285,226	280, 518	435.1503	C18H27O12	0.0	0.0	5.5	0.3
48	Loganic acid^*^	1.18	1.04	0.92	8.31	376	375	[M-H]^−^	375, 336, 329, 227, 144	280	375.1267	C16H23O10	-2.4	-6.4	5.5	0.2
49	Morusimic acid C	0.64	0.56	0.46	9.64	491	492	[M+H]^+^	492, 474, 456, 372, 330, 312, 268	254, 352	492.3181	C24H46NO9	0.8	1.6	2.5	1.3
50	Dicaffeoylquinic acid^*^	0.11	0.73	1.28	10.29	516	515	[M-H]^−^	515, 353, 286	280, 366	515.1182	C25H23O12	-0.8	-1.6	14.5	1.7
51	Cudracuspiphenone B^*^	0.49	1.82	1.50	17.27	312	311	[M-H]^−^	311, 296, 256, 183, 175	280	311.092	C18H15O5	0.1	0.3	11.5	0.1

* *Differential metabolites were selected by using the VIP value (>1.0) and p-value (< 0.05) from the partial least squares-discriminant analysis model in Figure 2*.

a *VIP, variable in projection; bRT, retention time; cMW, molecular weight*.

### Differences in physicochemical characteristics and anti-oxidant activities in 6 edible berries

Comparison of physicochemical characteristics, anti-oxidant activities, and contents of total phenolic, total flavonoid, and total anthocyanin among the 6 edible berries were examined to evaluate the palatability and quality characteristics of berries. The physicochemical characters sugar content, titratable acidity, and pH were measured (Table 3). The observed average sugar contents were similar among honeyberry, blueberry, mandarin melonberry, mulberry, and chokeberry, but relatively higher than that in Korean black raspberry. The pH and titratable acidity indicates the relative acidity and flavors of the berries. According to results, the relative acidity among berries was as follows: honeyberry (L) >> Korean black raspberry (R) > blueberry (V) = chokeberry (A) ≥ mulberry (M) >> mandarin melonberry (C). Particularly high acidity is observed in honeyberry, while low acidity is in mandarin melonberry.

**Table 3:** Physicochemical characters of 6 kinds of edible berries.

**Berry species**	**Sugar content (^°^Brix)**	**Titratable acidity (% acid)**	**pH**	**Total phenolic content (GEC, μg/mL)**	**Total flavonoid content (NEC, μg/mL)**	**Total anthocyanin content (CEC, μg/mL)**
Honeyberry	15.40 ± 1.58^a^	5.97 ± 0.67^a^	3.07 ± 0.06^a^	99.06 ± 34.09^a^	51.86 ± 24.24^a^	122.76 ± 57.65^a^
Blueberry	13.12 ± 1.50^ab^	1.02 ± 0.39^b^	3.39 ± 0.23^b^	51.81 ± 14.63^bc^	27.96 ± 10.03^bc^	82.97 ± 30.27^a^
Mandarin Melonberry	16.29 ± 2.52^a^	0.20 ± 0.08^c^	6.50 ± 0.23^c^	31.16 ± 10.08^c^	17.92 ± 6.88^c^	3.00 ± 0.59^b^
Mulberry	15.84 ± 1.47^a^	0.71 ± 0.29^b^	5.03 ± 0.36^d^	77.67 ± 19.40^ab^	44.65 ± 16.52^ab^	118.52 ± 56.42^a^
Chokeberry	15.40 ± 2.21^a^	1.12 ± 0.37^b^	3.92 ± 0.23^e^	194.61 ± 24.28^d^	84.02 ± 12.49^d^	127.46 ± 31.00^a^
Korean Black Raspberry	11.02 ± 1.05^b^	1.87 ± 0.29^d^	3.80 ± 0.13^e^	144.71 ± 26.14^e^	80.57 ± 24.82^d^	282.55 ± 51.10^c^

*GEC, gallic acid equivalent concentration; NEC, naringin equivalent concentration; CEC, cyanidin-3-glucoside equivalent concentration*.

*Different letters in the table indicate significant difference by ANOVA followed by Duncan's multiple-range test*.

**Figure 3:**
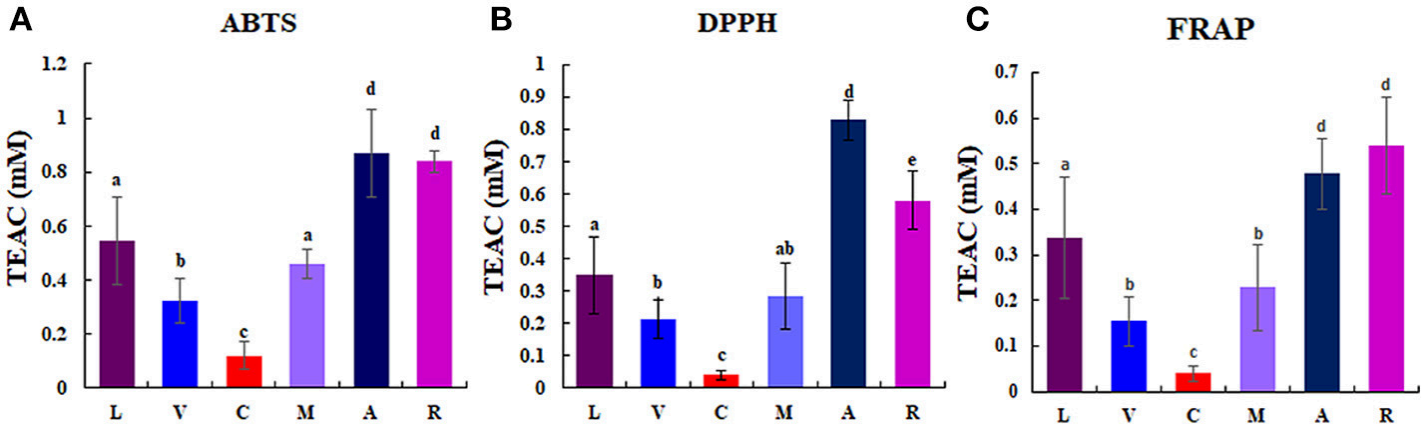
Results of anti-oxidant activities [ABTS **(A)**, DPPH **(B)**, FRAP **(C)**] in 6 different edible berries. Different letters in the bar graph indicate significant difference by ANOVA followed by Duncan's multiple-range test (*p* value < 0.05). TEAC: trolox equivalent antioxidant capacity. L, honeyberry; V, blueberry; C, mandarin melonberry; M, mulberry; A, chokeberry; R, Korean black raspberry.

Considerable differences of anti-oxidant activity were observed among berries as follows: chokeberry (A) ≥Korean black raspberry (R) >honeyberry (L) ≥mulberry (M) >blueberry (V) >mandarin melonberry (C) (Figure 3). About 10-fold anti-oxidant activities difference was showed between the highest (chokeberry and Korean black raspberry) and the lowest (mandarin melonberry). The total phenolic content and total flavonoid content levels of berries showed similar tendency with anti-oxidant activities (Table 3). However, the result of total anthocyanin content levels showed different distributions in berries as antioxidant activities as follows: Korean black raspberry (R) >honeyberry (L) = mulberry (M) = chokeberry (A) ≥blueberry (V) >mandarin melonberry (C).

These results demonstrated that the 6 different edible berries have unique phenotypes, physicochemical characteristics, and bioactivities. Through metabolic pathway analysis, we detected significantly different metabolites, explaining the differences in phenotypes among the berries.

### Comparison of metabolic pathway markers and bioactivity correlations in 6 edible berries

**Figure 4:**
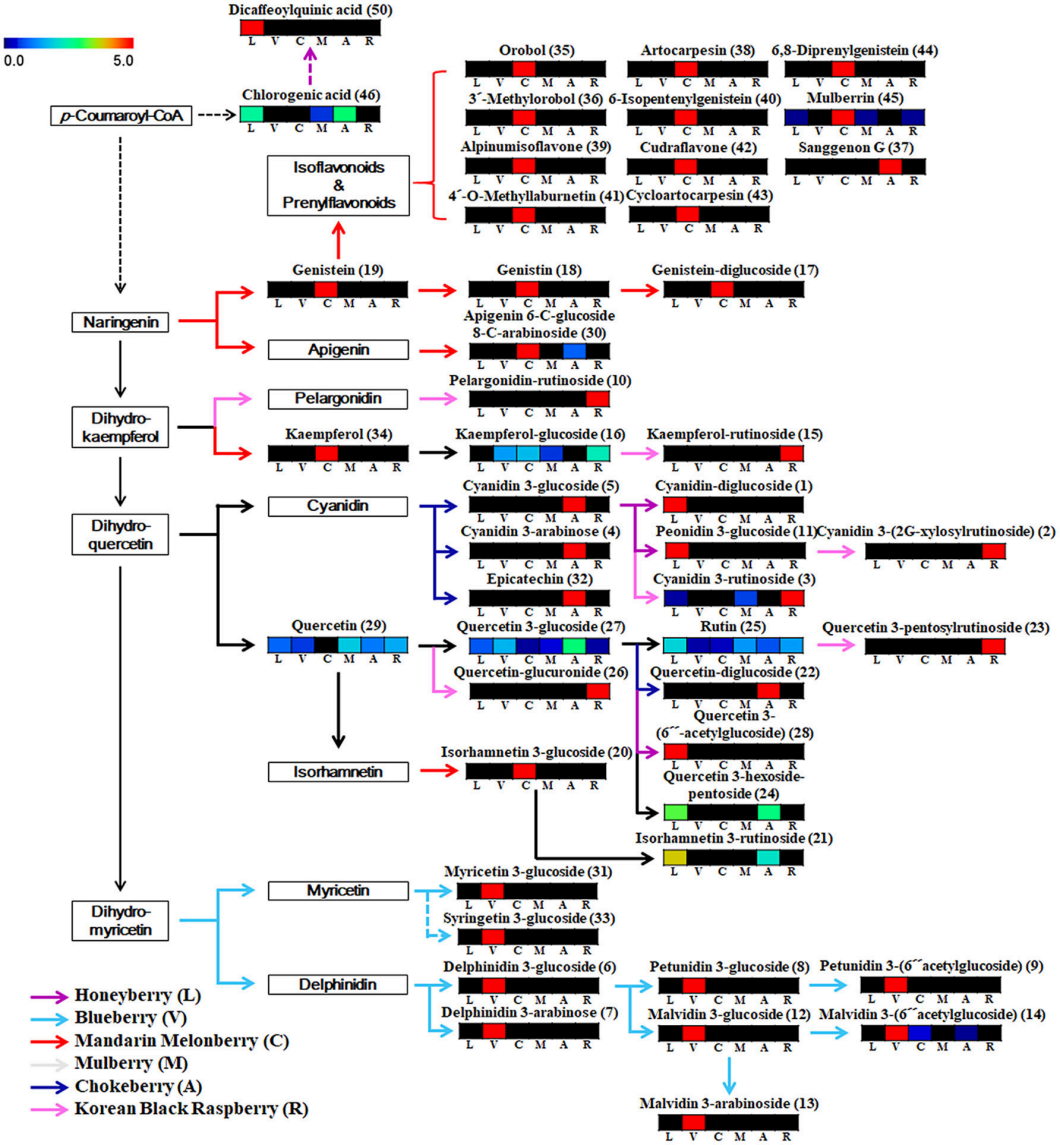
Schematic diagram of the biosynthetic pathway and relative content of metabolites in 6 different kinds of edible berries. The relative contents are presented as fold-changes normalized using the average of all values. The biosynthetic pathway was modified from the KEGG database (http://www.genome.jp/kegg/). L, honeyberry; V, blueberry; C, mandarin melonberry; M, mulberry; A, chokeberry; R, Korean black raspberry. Numbers on the biosynthetic pathway correspond to the metabolic numbers in Table 2.

Based on the list of identified metabolites, a secondary metabolite biosynthesis pathway of berries was proposed (Figure 4). To construct the biosynthetic pathways in different berries, we mostly referred to the KEGG (Kyoto Encyclopedia of Genes and Genomes) database and some literatures (Zifkin et al., 2012; Jaakola, 2013; Hyun et al., 2014). To evaluate the metabolic pathway markers of each berry, the relative average value of each metabolite was determined for the metabolic pathway, including biosynthesis of various anthocyanins and flavonoids. Anthocyanins and flavonoids, which were present in all berries examined, are synthesized through *p*-coumaroyl-CoA; however the biosynthetic routes of flavonoids and anthocyanins differed in each berry. Mandarin melonberry specifically contained several flavonoids biosynthesized from the intermediate precursor naringenin. These flavonoids were mainly composed of genistein (19), genistein glycosides (17, 18), and genistein-derived isoflavonoids and prenylflavonoids including orobol (35), 3′-methylorbol (36), alpinumisoflavone (39), 4′-o-methyllaburnetin (41), artocarpesin (38), 6-isopentenylgenistein (40), cudraflavone (42), cycloartocarpesin (43), 6,8-diprenylgenistein (44), and mulberrin (45). In addition, kaempferol (34) and isorhamnetin 3-glucoside (20) were specific to mandarin melonberry and are biosynthesized from the precursors dihydrokaempferol and isorhamnetin, respectively. Rutinoside contained flavonoids and anthocyanins such as dihydrokaempferol-derived pelagonidin-rutinoside (10) and kaempferol-rutinosie (15), dihydroquercein-derived cyanidin 3-rutinoside (3), cyanidin 3-(2G-xylosylrutinoside) (2), and quercetin 3-pentoxylrutinoside (23), which were synthesized specifically in Korean black raspberry. Anthocyanins, which are synthesized from the intermediate precursors dihydroquercetin and cyanidin, were abundant in chokeberry and honeyberry, except for the rutinoside forms; cyanidin 3-glucoside (5), cyanidin 3-arabinose (4), epicatechin (32) were high in chokeberry and cyanidin-diglucoside (1) peonidin 3-glucoside (11) were high in honeyberry. Metabolites biosynthesized from quercetin did not show high contents in certain types of berries but were detected in several berries simultaneously. Significantly high levels of anthocyanins, biosynthesized from dihydromyricetin, including myricetine 3-glucoside (31), syringetin 3-glucoside (33), delphinidin glycoside (6, 7), petunidin glycoside (8, 9), and malvidin glycoside (12, 13, 14), were observed in blueberry.

To evaluate the contribution of metabolites to anti-oxidant activities, we performed correlation analysis between significantly discriminate metabolites and bio-activities (Figure S1). Among them, several anthocyanin glycosides [cyanidin 3-(2G-xylosylrutinoside) and pelargonidin-rutinoside] and flavonoids (kaempferol-rutinoside, quercetin 3-pentosylrutinoside, and quercetin-glucuronide), which were specifically distributed in Korean black raspberry, showed a positive correlation with bio-activities. In addition, chokeberry specifically distributed metabolites (cyanidin 3-arabinoside, cyanidin 3-glucoside, quercetin-di-glucoside, quercetin 3-glucoside, epicatechin, and sanggenon G) and metabolites highly distributed in both chokeberry and honeyberry (isorhamnetin 3-rutinoside and quercetin 3-hexoside-pentoside) were highly correlated with bio-activities. Thus, consumption of various types of berries with different metabolite compositions may have overall nutritional benefits.

## Discussion

Recently, consumption of berries has rapidly increased because of their well-known high polyphenol contents (e.g., phenolic acids, flavonoids, and anthocyanins), which can prevent various diseases and disorders (Nile and Park, 2014). Many studies have examined these bio-activities such as for specific polyphenols by targeted analysis of a few berries (Wang et al., 2014a; Ramirez et al., 2015; Ancillotti et al., 2016). In this study, we performed non-targeted metabolite profiling of 6 different edible berries to compare the metabolite distributions and found differences in secondary metabolites biosynthesis routes among the berries. Our results agree with those of previous studies that compared the levels of anthocyanins, phenolics, and antioxidant capacity among different small berries (Moyer et al., 2002; Szajdek and Borowska, 2008). According to taxonomic classification (Figures 1B,C), the result of chemotaxonomic classification showed different patterns in the phylogenetic tree. These results indicate that various anthocyanins and flavonoids are positively correlated with anti-oxidant activities; however, different forms of anthocyanin and flavonoid in each berry type can reveal the unique antioxidant activities of various species. Berries are exceptionally rich sources of antioxidant polyphenols, but most metabolites showed different distribution patterns in each berry, as the major types of these metabolites varied among berries, which may be related to differences in the regulation of gene expression (Zifkin et al., 2012). Most of key regulatory genes and enzymes controlling anthocyanin and flavonoid biosynthetic pathway were well discovered in model plants such as grape berry (Wang et al., 2017), Arabidopsis (Lepiniec et al., 2006), and blueberry (Zifkin et al., 2012). However, those of genes in other kinds of berries are relatively not yet been investigated. According to genomic information of berries, 144 nucleotide sequences and 82 proteins from honeyberry (*Lonicera caerulea*); 319 nucleotide sequences and 135 proteins from blueberry (*Vaccinium corymbosum*); 45 nucleotide sequences and 24 proteins from mandarin melonberry (*Cudrania tricuspidata*); 4,386 nucleotide sequences and 686 proteins from mulberry (*Morus alba*); 47 nucleotide sequences and 101 proteins from chokeberry (*Aronia melanocarpa*); 141 nucleotide sequences and 17 proteins from Korean black raspberry (*Rubus coreanus*) have been deposited in NCBI database. Therefore, large disparity of genomic researches among berries were observed, and further researches are needed for fully understanding of anthocyanin and flavonoid biosynthetic pathway in berry. Comparative metabolomics could provide different anthocyanin and flavonoid compositions in biosynthetic pathway of each berry, which could be an important information for connection with genomic research.

Blueberry (*V. corymbosum*) is the most popular berry species and has been widely studied to determine its polyphenol profiles, bio-activities (Heinonen, 2007; Castrejón et al., 2008; Lee et al., 2014), and related gene expression (Zifkin et al., 2012). This species is also known to have high anti-oxidant activity, but showed average anti-oxidant activities among the 6 edible berries evaluated in this study (Figure 3). Various anthocyanin glycosides derived from an intermediate precursor, dihydromyricetin, including myricetin-, syringetin-, delphinidin-, petunidin-, and malvidin-glycosides were specifically higher in blueberry than in other berries (Figure 4). Specific anthocyanin accumulation can be explained by gene expression of related genes such as flavonol synthase, anthocyanidin synthase, UDP-Glc:flavonoid-3-*O*-glycosyltransferase, and anthocyanin-*O*-methyltransferase, which are expressed during the development of blueberry fruit (Zifkin et al., 2012).

According to the results of multivariate statistical analysis, chokeberry was clearly separated from the other berries in the PCA score plot (Figure 2A). In terms of bio-activities, chokeberry showed the highest anti-oxidant activity and an average sugar content, titratable acidity, and pH among the berries. Chokeberry contained higher levels of cyanidin-derived cyanidin monoglycoside and epicatechin than other berries according to biosynthetic pathway analysis (Figure 4), which may be related to its high anti-oxidant activity. This is consistent with the results of a previous study (Szajdek and Borowska, 2008). Differentially accumulated anthocyanin forms show large discrepancies in antioxidant capacity. Zheng and Wang reported that chokeberry (*Aronia* species) has significantly higher anti-oxidant activities than blueberry (*Vaccinium* species), and this variation was related to the distribution of phenolic compounds according to the relatively high levels of cyanidin monoglycosides and caffeic acid derivative in chokeberry and various dihydromyricetin-derived anthocyanin glycosides in blueberry (Zheng and Wang, 2003). However, further studies are needed to confirm these secondary metabolite distributions in berries with related gene expression.

The anti-oxidant capacity of Korean black raspberry was similar to that of chokeberry species. According to our results and another report, higher anti-oxidant activity is affected by high concentrations of total phenolic, flavonoids, and anthocyanins (Figure 3, Table 3; Choi and Kwak, 2014). In Korean black raspberry, particularly high contents of anthocyanin-rutinoside derivatives [pelargonidin-rutinoside, cyanidin 3-rutinoside, and cyanidin 3-(2G-xylosylrutinoside)] and some flavonoid-rutinoside derivatives (kaempferol-rutinoside and quercetin 3-pentosylrutinoside) were observed. Identical metabolite distribution patterns were observed in another study (Tian et al., 2006; Veberic et al., 2015) and related gene expression was confirmed (Hyun et al., 2014). *Rubus coreanus* chalcone isomerase 2 was shown to increase the levels of anthocyanin rutinosides in Korean black raspberry (*R. coreanus*) by *Arabidopsis* complementation analysis. Additionally, up-regulation of *F3*′*3-, DFR4*, and *LDOX1* was observed during fruit ripening (Hyun et al., 2014). Furthermore, Korean black raspberry showed the lowest sugar content and relatively high titratable acidity among berries. According to a previous report, the sweet tastes and flavor of berry fruit is affected by not only high sugar contents, but also low levels of organic acids. The lowest sugar contents in Korean black raspberry were verified not only among the 6 edible berries in this study, but also among 25 wild or cultivated berry species reported previously (Mikulic-Petkovsek et al., 2012a).

Most flavonoids derived from naringenin were identified by mandarin melonberry-specific detection by non-targeted metabolite profiling, except for sanggenon G and apigenin 6-C-glucoside 8-C-arabinoside (Figure 4). Mandarin melonberry contained high levels of various flavonoids other than anthocyanins, such as prenylated flavonoids and isoflavonoids (Shin et al., 2015; Suh et al., 2016). Prenylated flavonoids are synthesized by *C. tricuspidata* isoliquiritigenin 3′-dimethylallyltransferase, flavonoid prenyltransferases, which catalyze the prenylation of several chalcones (Wang et al., 2014b). According to its physicochemical characteristics, mandarin melonberry has a sweet taste possibly because of its high levels of sugar and low contents of titratable acidity compared to other berries (Table 3). Furthermore, this berry showed the lowest anti-oxidant activities. According to previous reports, the anti-oxidant activity of flavonoids depends on the nuclear structures and types of substitutions. Several studies reported that most of anthocyanins have higher anti-oxidant capacities than other flavonoids because their flavylium cationic structure has several hydroxyl groups and different substituents in ring B at low pH (Rice-Evans et al., 1996; Heim et al., 2002; Yan et al., 2002; Zhang et al., 2008).

Honeyberry and mulberry showed similar patterns in bio-activities and physicochemical characteristics, including anti-oxidant activities, sugar content, total phenolic content, total flavonoid content, and total anthocyanin content. Particularly, honeyberry had the highest acidity among the berries. High organic acid levels accumulated during the fruit ripening of honeyberry, which may be related to its high acidity and sour taste (Lee et al., 2015). In the biosynthetic pathway, similar to chokeberry, dihydroquercetin-derived anthocyanins were detected in honeyberry, but no identical compounds were found.

In contrast, no specific anthocyanins or flavonoids synthesis routes were found in mulberry. Several metabolites including quercetin, quercetin 3-glucoside, rutin, chlorogenic acid, and kaempferol-glucoside were identified in the metabolic pathway of mulberry, but with no significantly high levels among berries. Mulberry is relatively unknown compared to other berries. Few studies have examined mulberry and its bio-active compounds. Several studies reported that mulberry contains pyrrole alkaloids as major secondary metabolites (Asano et al., 2001; Kim et al., 2013). In our results, morusimic acid C, a pyrrole alkaloid compound, showed specifically high content in mulberry (Figure S2). However, no alkaloid compounds other than morusimic acid C were found in this study. This may be because of differences in the structure and chromatographic behaviors between flavonoids and alkaloids (He, 2000; Ding et al., 2007). To identify the unique characteristics of mulberry species from among other berries, additional studies are needed.

## Conclusion

In this study, we performed metabolite comparisons of 6 different edible berries to examine their biosynthetic pathways using non-targeted metabolite profiling and measured the anti-oxidant activity and physicochemical parameters to explain the different characters of each berry. We demonstrated that each berry has different anthocyanin and flavonoid compositions by biosynthetic pathway analysis and that these differences affected the nutritional benefits of berries. This biosynthetic pathway-based non-targeted metabolite profiling improves the understanding of differences in the metabolite distribution among berry species without bias and provides correct ingredients information for potential applications of important plants. Further studies focusing on robust multi-omics approaches are required to fully understand the biosynthesis of secondary metabolite differences among berries.

## Author contributions

CL supervised and took complete responsibility for this project. DS performed data processing, statistical analysis, and wrote the article. GL measured the physicochemical characters and MS analysis. EJ discussed the results and wrote the article.

### Conflict of interest statement

The authors declare that the research was conducted in the absence of any commercial or financial relationships that could be construed as a potential conflict of interest.

## Supplementary material

Figure S1 Correlation map analysis between bio-activities and significantly discriminant metabolites in 6 different kinds of edible berries.

Figure S2 Heatmap of 6 different types of edible berries derived from UHPLC-LTQ-IT-MS/MS data. L, honeyberry; V, blueberry; C, mandarin melonberry; M, mulberry; A, chokeberry; R, Korean black raspberry.
