# Supplementary figures and images for: Distinguishing Six Edible Berries Based on Metabolic Pathway and Bioactivity Correlations by Non-targeted Metabolite Profiling
Source: Front Plant Sci. 2018 Oct 2;9:1462. doi: 10.3389/fpls.2018.01462 (PMC6175979; doi:10.3389/fpls.2018.01462)

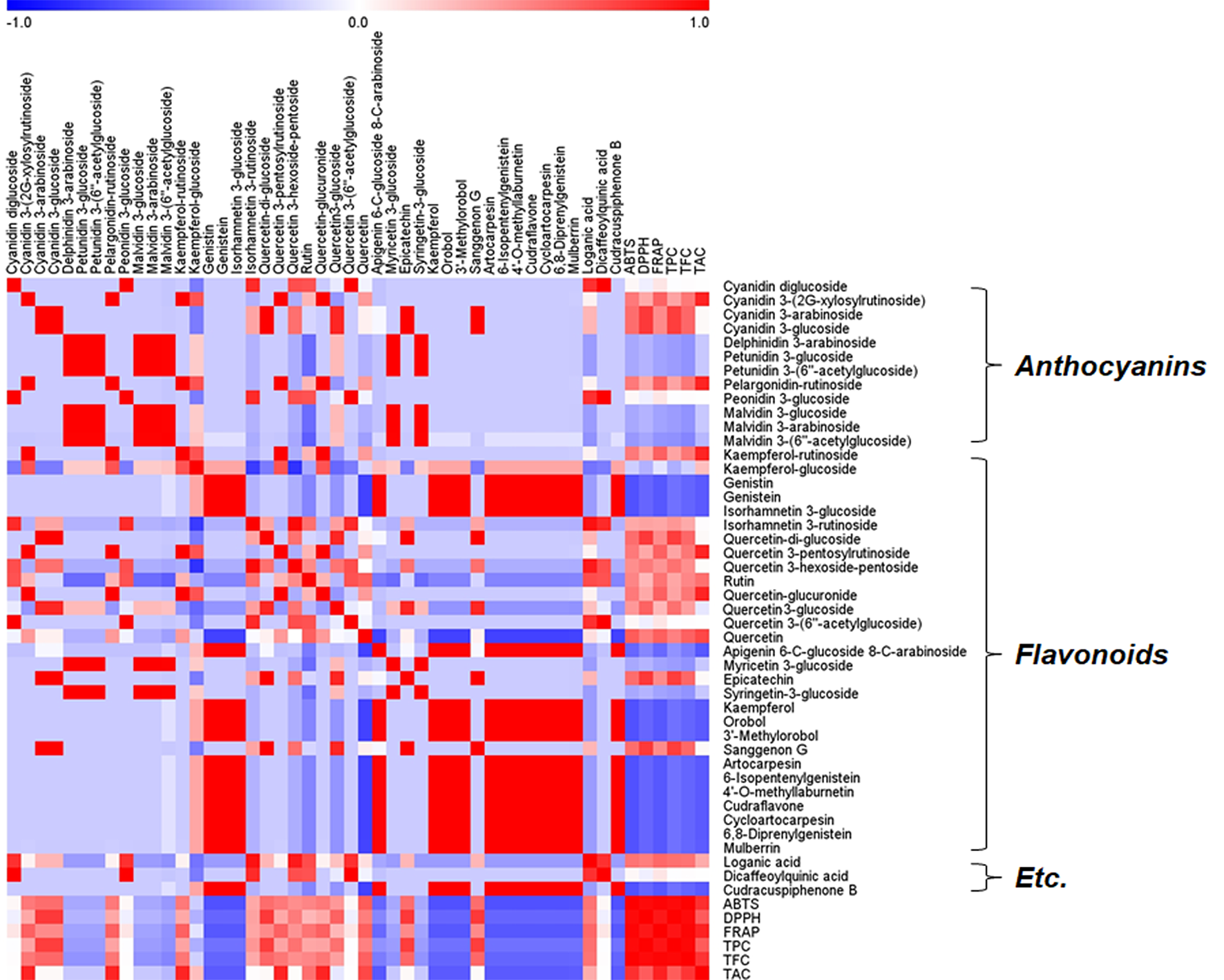

Supplement: Figure S1 — Correlation map analysis between bio-activities and significantly discriminant metabolites in 6 different kinds of edible berries. [file Image_1.TIF]

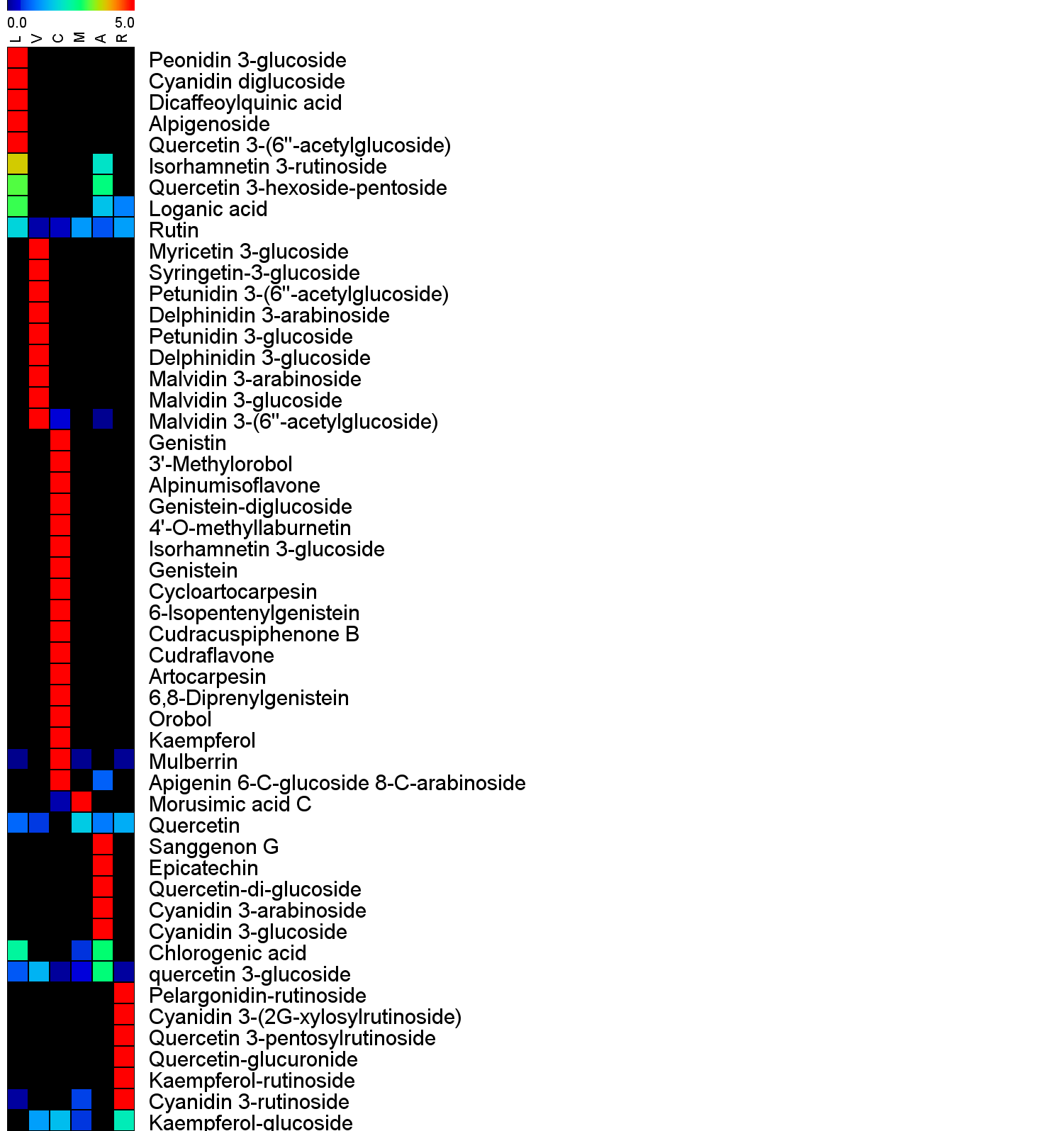

Supplement: Figure S2 — Heatmap of 6 different types of edible berries derived from UHPLC-LTQ-IT-MS/MS data. L, honeyberry; V, blueberry; C, mandarin melonberry; M, mulberry; A, chokeberry; R, Korean black raspberry. [file Image_2.JPEG]
